# Dietary Patterns, Blood Pressure and the Glycemic and Lipidemic Profile of Two Teenage, European Populations

**DOI:** 10.3390/nu13010198

**Published:** 2021-01-10

**Authors:** Maria Kafyra, Ioanna Panagiota Kalafati, Satish Kumar, Maria Spyridoula Kontoe, Christine Masson, Sophie Siest, George V. Dedoussis

**Affiliations:** 1 Department of Nutrition and Dietetics, School of Health Science and Education, Harokopio University, 17671 Athens, Greece or mariakaf@hua.gr (M.K.); nkalafati@gmail.com (I.P.K.); 2 IGE-PCV, Université de Lorraine, F-54000 Nancy, France; satish.kumar@univ-lorraine.fr (S.K.); maria.spyridoula.kontoe@gmail.com (M.S.K.); christine.masson@univ-lorraine.fr (C.M.); sophie.visvikis-siest@inserm.fr (S.S.)

**Keywords:** dietary patterns, teenagers, European populations, blood pressure, glucose, cholesterol, triglycerides, cardiometabolic risk factors

## Abstract

The present study sought to retrospectively investigate the dietary habits of two adolescent, European populations from the cross-sectional Greek TEENAGE Study and French STANISLAS Family Study. We aimed to explore the relation between the populations’ dietary patterns and blood pressure, glycemic and lipidemic profile. Dietary patterns were extracted via Principal Component Analysis (PCA), based on data collected from two 24 h dietary recalls for the TEENAGE study and a 3-day food consumption diary for the STANISLAS study. Multiple linear regressions and mixed models analyses, adjusting for confounding factors, were employed to investigate potential associations. A total of 766 Greek teenagers and 287 French teenagers, were included in analyses. Five dietary patterns were extracted for each population accounting for 49.35% and 46.69% of their respective total variance, with similarities regarding the consumption of specific food groups (i.e., western-type foods). In the TEENAGE Study, the “chicken and sugars” pattern was associated with lower CRP levels, after adjusting for confounding factors (*p*-value < 0.01). The “high protein and animal fat” dietary pattern of the STANISLAS Family Study was related to higher BMI (*p*-value < 0.01) and higher triglycerides levels (*p*-value < 0.01). Our findings summarize the dietary habits of two teenage, European populations and their associations with cardiometabolic risk factors.

## 1. Introduction

Adolescence constitutes a period of increased nutritional needs, required to support the physical growth that accompanies puberty [1,2]. Healthy eating is of vital importance during adolescence [3,4], in order to ensure the sufficient macronutrient and micronutrient intake needed for proper physical development [1], cognitive performance [5,6,7] and good mental health [8]. Dietary habits during the adolescent years directly influence body weight regulation and play a major role in the healthy development that comes with adolescence [9]. Adherence to “unhealthy” eating habits during this period increases the risk of obesity development [10,11], which has, in turn, been long associated with an increased risk of non-communicable disease manifestation, such as type 2 diabetes, both in adolescence and later on in adult life [9,10]. Indeed, the presence of adolescent obesity has been associated with severe obesity in late adulthood [12,13] and a greater risk for type 2 diabetes development in early adulthood [13]. In addition, higher Body Mass Index (BMI) values during adolescence have been associated with higher BMI values during adulthood, as well as a 30 to 40% increased risk in adult mortality [14]. 

The causes of overweight, obesity and non-communicable disease development in adolescent populations are related to the consumption of energy-dense foods, reduction of physical activity, as well as socioeconomic factors, such as food availability and food preference, influenced by geographic factors [9,11]. Energy-dense foods have been related both directly and indirectly, via their positive association, with overweight and obesity development, in the development of non-communicable diseases [11]. Indeed, poor eating habits have regularly been associated with a high consumption of foods with high fat and/or sugar contents [9]. 

Adolescent dietary habits are also directly linked to the teenagers’ metabolic profile and the interplay between biomarkers of glycemic and lipidemic control [15]. It has been shown that adherence to an “unhealthy” dietary pattern is associated with a higher risk for metabolic syndrome presence [15]. Their importance is further highlighted by the increased incidence of type 2 diabetes in young children and teenagers [16]. Consumption of energy-dense foods in children and teenagers with a family history of type 2 diabetes, plays a central role in the formation of a worse glycemic profile and potentially, subsequent development of type 2 diabetes (T2D) [16]. T2D in children is associated with a deteriorated lipidemic profile (i.e., dyslipidemia), as a direct effect of the observed insulin resistance [17]. A different study showed that Greek children with dyslipidemia and unfavourable dietary habits, such as consuming only one meal per day, displayed higher levels of various biomarkers of lipidemic control, namely total cholesterol and low-density lipoprotein cholesterol (LDL-C) [18].

Another cardiometabolic risk factor receiving more and more attention is the development of hypertension and the elevated levels of arterial pressure in adolescents. Indeed, high blood pressure can be met in teenagers, with boys reporting higher levels of blood pressure than girls [19].

The present analyses constitute the first step in the context of the 2018 Gutenberg Chair project, aimed at firstly investigating the role of dietary habits in the anthropometric and biochemical profile of two adolescent, European populations and subsequently exploring the potential role of nutrition as a modifier of genetic make-up in adolescence. The latter will take place via an investigation into the relationship between the populations’ dietary habits and their glycemic and lipidemic profile and inflammation markers with genetic risk scores created for anthropometric indices, biomarkers of glycemic and lipidemic control and inflammation markers.

In this context, the aim of the present study is to investigate the dietary habits of the two populations from the Greek TEENAGE Study and the French STANISLAS Family study and their potential associations with blood pressure, biomarkers of glycemic and lipidemic control and levels of C-reactive protein (CRP). Therefore, the objectives of the study are formed as follows: (a) to identify the dietary patterns of adolescents in the Greek and French cohorts; and (b) to investigate potential, respective associations between said patterns and blood pressure, anthropometric indices, biomarkers of glycemic and lipidemic control and CRP levels.

## 2. Materials and Methods 

### 2.1. The TEENAGE Study Cohort

The TEENAGE (TEENs of Attica: Genes and Environment) study constitutes a cross-sectional study conducted during the period 2008–2010 in the region of Attica, Greece [20,21]. The study was approved by the Institutional Review Board of Harokopio University and the Greek Ministry of Education and Religious Affairs and took place adhering to the guidelines of the Declaration of Helsinki [22]. The study consisted of a sample of healthy, Greek adolescent students residing in the Attica region during the period of recruitment.

All students and their parents received written information on the aims and the procedures of the study prior to enrolment and all participants provided written consent. All students enrolled participated in an assessment session with either a nutrition or a pediatric health-care professional, which included clinical examination, collection of blood samples, conduct of a 24 h dietary and physical activity recall and collection of anthropometric and lifestyle data. A second 24 h dietary and physical activity recall was conducted via telephone, 3 to 10 days after the in-person meeting. Overall, data for an original sample of 857 adolescent students from 1440 schools in the region of Attica, aged 13 to 15 years old, were cross-sectionally collected.

Collection of anthropometric data during the in-person meeting consisted of height (measured to the nearest 0.1 cm), weight (measured to the nearest 0.1 kg), waist and hip circumference and skinfold measurements (measured to the nearest 0.1 mm). Height was measured using a portable stadiometer, where participants were barefoot, looking ahead and with relaxed shoulders. Weight was measured via use of scales, where participants were barefoot and with light clothing. BMI was calculated as weight divided by height (kg/m^2^). Waist and hip circumference were measured using a soft tape, the former between the twelfth rib and the iliac crest and the latter at the widest point of the hips. Two skinfold measurements were collected for each of the triceps, subscapular and suprailiac skinfolds, using the Lange skinfold calipers. 

Assessment of dietary habits took place via the collection of the two non-consecutive 24 h dietary recalls, which were conducted on different days of the week. Analysis of the data collected took place via use of the Nutritionist Pro software, version 2.2 [23]. The ratio of reported energy intake to BMR was calculated for each student, in order to assess potential under-reporting. BMR was calculated using the Schofield equations [24,25] and cut-off points [26] were adapted to the ones reported for children and adolescents [27]. Participants who had previously reported dieting in the past or never dieting, were excluded.

For the purposes of the present study, we used the available anthropometric, biochemical and dietary data of 766 adolescent students (as shown in Table 1). Dietary pattern extraction was based on the mean consumption of food groups, derived from the two non-consecutive 24 h dietary recalls. 

### 2.2. The STANISLAS Family Study Cohort

The STANISLAS (Suivi Temporaire Annuel Non Invasif de la Sante des Lorrains Assures Sociaux) Family Study constitutes a cross-sectional study conducted during the period 1993–1995 in the region of Vosges and the South of Meurthe and Moselle (East part of France) [28,29]. The study was approved by the advisory committee for the protection of people in biomedical research in Nancy, France. The study consisted of a sample of nuclear families with parents aged up to 65 years old and children older than 6 years at the time of recruitment, residing in the aforementioned region. The study only included families with healthy family members, reporting no comorbidities and/or chronic diseases, residing in the aforementioned regions at the time of recruitment. Willing participants residing in the region of Nancy further participated in 5-year follow-ups up to the period 2003–2005 [30].

All included families provided informed consent. The families enrolled participated in an in-person session with trained professionals, which included clinical examination, collection of blood samples and collection of anthropometric, dietary and lifestyle data. Collection of the food-related surveys was conducted by dietitians. Blood pressure, pulse rate, skinfold thickness and bone density were measured by nurses and pubertal development and family history of cardiovascular diseases was assessed by general practitioners. Data on alcohol and tobacco consumption, physical activity, education and socio-professional status were collected through questionnaires, under the supervision of trained nurses. Overall, data for an original sample of 1006 families were cross-sectionally collected. 

Weight, height, waist-to-hip ratio and impedancemetry measurements were conducted by technical operators. BMI was, again, calculated as weight divided by height (kg/m^2^). Assessment of dietary habits took place via collection of a 3 day food consumption diary, for two continuous days within the week and one day of the weekend. Analysis of the data took place via use of the GENI package, nutritional database program [31]. 

For the purposes of the present study, we used the available anthropometric, biochemical and dietary data of 287 adolescents at the time of the baseline recruitment (as shown in Table 2). Dietary pattern extraction was based on the mean consumption of food groups, deriving from the 3 day food consumption diary. Low-density cholesterol (LDL-C) for this cohort was calculated based on the available data for total cholesterol (TC), high-density lipoprotein cholesterol (HDL- C) and triglyceride (TG) levels, using the Friedeweld Equation, as follows [32]:

LDL − C = (TC) − (HDL − C) − (TG/5)

### 2.3. Statistical Analysis 

The entirety of the data handling and data analyses was carried out using the SPSS Software [33]. Body Mass Index (BMI) was calculated as weight divided by height (kg/m^2^). Assessment of the variables’ distribution was conducted via use of the Shapiro–Wilk test, demonstrating the mean and standard deviation for all normally distributed variables and the median and interquartile range for all variables not following the normal distribution (Shapiro–Wilk *p*-value > 0.05). We used the Student’s *t*-test and Mann–Whitney test for all hypotheses testing for continuous variables. 

We performed Principal Component Analyses (PCA) in order to extract all dietary patterns for both populations [34]. PCA constitutes an epidemiological tool, largely used in the assessment of dietary data and the subsequent extraction of dietary patterns [35], having been previously tested in large young populations [36]. PCA was conducted on 15 food groups for the TEENAGE study population and 15 food groups for the STANISLAS Family study population, based on the available data for the cohorts. 

The Kaiser–Meyer–Olkin (KMO) test was calculated at 0.545 and 0.576 for the TEENAGE and the STANISLAS teenagers, respectively, indicating mediocre to sufficient data adequacy. The varimax orthogonal rotation was used for the extraction of the patterns and the Kaiser criterion was set at retaining 5 components with Eigen values bigger than 1.

We further tested for potential associations between the extracted dietary patterns, blood pressure and biomarkers of glycemic and lipidemic control, as well as levels of CRP, via use of multiple linear regressions in the TEENAGE cohort and linear mixed models in the STANISLAS cohort. Given that the STANISLAS Family Study consisted of a cohort of families, we used the latter in order to correct for the potential familial bias of siblings included in the analyses [37,38]. We classified the different siblings of each family as the repeated measures, compound symmetry as the repeated covariance type and all adjusting factors and dietary patterns as the fixed effects. Potential associations were investigated, adjusting for 3 different models of confounding factors. Model 1 included adjustment solely for the age and sex of the participants; Model 2 included adjustment for sex, age and level of physical activity; Model 3 consisted of adjustment for their age, sex, level of physical activity and BMI; and, finally, Model 4 included adjustment for age, sex, physical activity, BMI and energy intake. All tested variables were log-transformed. Multiple linear regression results are presented as beta coefficients (β) and standard error (SE). Linear mixed model results are presented as estimates and standard error (SE). All statistical analyses included the level of nominal significance set at α = 0.05. The adjusted threshold after multiple testing was set to (0.05/5 components examined, i.e., dietary patterns = 0.01).

## 3. Results

### 3.1. Descriptive Characteristics

The anthropometric and biochemical characteristics of the two populations are depicted in Table 1 and Table 2. Concerning the TEENAGE cohort, a total of 766 teenagers (45.56% boys, 54.43% girls), with a median age of 13.30 years, were included in the analyses. The STANISLAS cohort provided data for 287 teenagers (47.73% boys, 525.26% girls), with a median age of 13.08 years.

**Figure 1:**
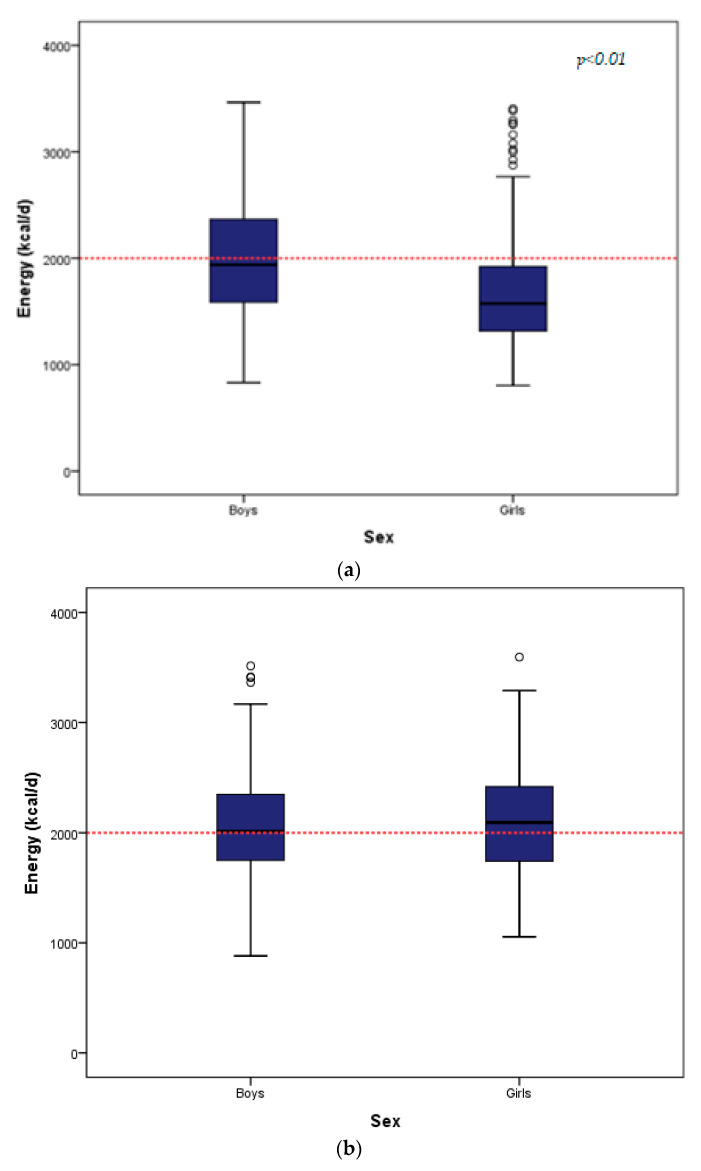
(**a**) Boxplot of daily caloric intake in the TEENAGE study. (**b**) Boxplot of daily caloric intake in the STANISLAS Family Study.

The daily energy intake for the two populations by sex, is depicted in Figure 1. As shown in Table 1 and Table 2, the Greek teenagers reported a median energy intake of 1741.00 kcal/d (IQR = 760), significantly different between the two genders, with boys reporting a higher intake. The French teenagers reported a median energy intake of 2056.03 kcal/d (IQR = 662.24), without presenting significant differences between sexes. 

### 3.2. Extraction of Dietary Patterns 

PCA for the TEENAGE cohort resulted in the identification of 5 dietary patterns, accounting for 49.35% of the sample’s total variance. Food groups’ factor loadings in the respective patterns are presented in Table 3. 

The presented factor loadings depict each food group’s highest contribution and subsequent inclusion in one out of the five patterns (components) highlighted. Therefore, the dietary patterns formed are the following: (a) a “western breakfast” dietary pattern, consisting of cheese, dairy and processed meat, accounting for the highest percentage of the individual variance explained (15.61%); (b) a “legumes and good fat” pattern, including high consumption of legumes, olives, olive oil and nuts and accounting for 10.32% of the variance explained; (c) a “homemade meal” pattern, referring to high consumption of red meat and potatoes, associated with lower fish consumption and explaining 8.33% of the total variance; (d) a “chicken and sugars” pattern, including high consumption of chicken and sweets, associated with lower the consumption of fruits and juices, with a 7.60% of the variance explained; and (e) a “eggs and fibers” pattern, comprising of high consumption of non-refined cereals, vegetables and eggs, associated with lower refined cereals’ consumption and explaining 7.47% of the total variance.

PCA for the STANISLAS cohort resulted in the identification of 5 dietary patterns accounting for 46.69% of the sample’s total variance. Food groups’ factor loadings in the respective patterns are presented in Table 4. 

In a similar way to the aforementioned, the presented factor loadings depict each food group’s highest contribution and subsequent inclusion in one out of the five patterns (components) highlighted. Therefore, the dietary patterns formed for this cohort are the following: (a) a “western breakfast” dietary pattern, consisting of cheese, breads and flours, processed meat and vegetables and accounting for the highest percentage of the individual variance explained (10.58%); (b) a “prudent snacking” pattern, including high consumption of eggs and vegetable fats, lower consumption of salty snacks and accounting for 10.44% of the variance explained; (c) a “high protein and animal fat” pattern, referring to consumption of red meat, animal fat and milk and yogurt, explaining 9.26% of the total variance; (d) a “fish and seafood” pattern, including high consumption of fish and seafood and lower consumption of poultry, with a 8.19% of the variance explained; and (e) a “sugary snacks” pattern, comprising of consumption of soft drinks, sugars, sweets and cereal bars and explaining 8.19% of the total variance. 

### 3.3. Multiple Linear Regressions in the TEENAGE Study

The multiple linear regressions adjusted for the three models of confounding factors, as described above, are shown in Table 5. Based on the available data, we examined associations between the patterns and the log-transformed values for BMI, WHR, SBP, DBP, glucose, insulin, HOMA-IR, TC, HDL- C, LDL- C, TG and CRP levels. The “legumes and good fat” pattern was associated with lower values of logBMI (β = −0.006, *p*-value = 0.017) and logInsulin (β = −0.020, *p*-value = 0.030), after the adjustments of Model 1. The “homemade meal” pattern was associated with lower values of logBMI (β = −0.005, *p*-value = 0.042), adjusting for Model 1. The “chicken and sugars” pattern was slightly associated with logGlucose Model 1 (β = 0.015, *p*-value = 0.017). The same pattern was associated with lower values of logInsulin after adjusting for Model 1 (β = −0.020, *p*-value = 0.030), Model 3 (β = 0.018, *p*-value = 0.049) and Model 4 (β = 0.018, *p*-value = 0.041). Moreover, the latter was further associated with lower values of logCRP in all models (Model 1: β = −0.051, *p*-value = 0.006, Model 2: β = −0.057, *p*-value = 0.004, Model 3: β = −0.050, *p*-value = 0.008, Model 4: β = −0.051, *p*-value = 0.008). No associations were found between the “eggs and fibers” pattern and the variables in all models. Statistically significant associations after assessment of the adjusted threshold were only maintained for the “legumes and good fat” pattern and the “chicken and sugars” pattern and logCRP in all models.

### 3.4. Linear Mixed Models in the STANISLAS Family Study

The linear mixed models adjusted for the three models of confounding factors, as described above, are shown in Table 6. Based on the available data, we examined associations between the patterns and the log-transformed values for BMI, WHR, SBP, DBP, glucose, TC, HDL-C, LDL-C, TG and CRP levels. The “western breakfast” pattern was associated with lower values of logCRP, in Model 4 (est = −0.076, *p*-value = 0.024). The “high protein and animal fat” pattern was associated with higher values of logBMI after adjustment for Models 1 and 2 (est = 0.011, *p*-value = 0.002, est = 0.009, *p*-value = 0.020), lower values of logDBP adjusting for Models 3 and 4 (est = −0.010, *p*-value = 0.045, est = −0.012, *p*-value=0.028, respectively) and higher values of logTriglycerides in all models (Model 1: est = 0.054, *p*-value < 0.001; Model 2: est = 0.049, p-value = 0.001; Model 3: est = 0.045, *p*-value = 0.002, Model 4:est = 0.041, *p*-value = 0.009) The “fish and seafood” pattern was associated with lower logDBP values (est = 0.009, *p*-value = 0.039), in Model 1. The “sugary snacks” pattern was associated with lower values of logHDL-C (est = −0.014, *p*-value = 0.049) in Model 3. No associations were found between the “prudent snacking” pattern and the variables in all models. Statistically significant associations after assessment of the adjusted threshold were only maintained for the maintained for the “high protein and animal fat” pattern and logBMI, in Model 1, as well as logTriglycerides in all models. Table 5. Linear Regression Analyses on the association between the dietary patterns, anthropometric indices and biomarkers of glycemic and lipidemic control in the TEENAGE study.

## 4. Discussion

The present study sought to investigate the dietary patterns of two adolescent, European populations, based on data from the Greek TEENAGE and the French STANISLAS Family studies, as well as their potential relations with blood pressure, biomarkers of glycemic and lipidemic control and levels of CRP. The study includes healthy teenagers from the two European populations, with a median BMI of 20.88 kg/m^2^ (IQR = 5.88 kg/m^2^) and 18.44 kg/m^2^ (IQR = 3.61 kg/m^2^). For the Greek teenagers, weight, waist-to-hip ratio (WHR), systolic blood pressure (SBP), levels for glucose, HOMA-IR, insulin, HDL-C and CRP significantly differed between boys and girls. Boys presented slightly higher values for weight, WHR, SP and glucose levels, while girls reported slightly higher levels of HOMA-IR, insulin and HDL-C. In the French teenagers group, WHR, SBP, glucose and total cholesterol levels presented statistically significant differences between the two sexes, with boys reporting slightly higher values for WHR, SBP and glucose levels and girls for total cholesterol levels. The teenagers of the study were mostly normal weighted. Both populations reported a mediocre energy intake (TEENAGE: 1741.00 kcal/d and STANISLAS: 2056.03 kcal/d), based on the present dietary guidelines for adolescents [39]. This could explain the fact that teenagers of both populations mostly reported BMI values within the normal range (18.5–25 kg/m^2^).

Five dietary patterns were identified in each population. The Greek “eggs and fibers” and the French “prudent snacking” patterns, explaining 7.47% and 10.44% of the respective total variance, included consumption of Mediterranean diet-related food groups, such as non-refined cereals, vegetables and eggs in the Greek teenagers and consumption of eggs and vegetable fats in French adolescents. The Greek teenagers showed a preference for healthy and traditional food combinations, such as consumption of legumes, olives, olive oil and nuts in the “legumes and good fat” pattern and consumption of red meat and potatoes in the “homemade meal” pattern, respectively. The French teenagers opted for consumption of more energy-dense food groups, such as red meat, animal fat and milk and yogurt in the “high protein and animal fat” pattern and soft drinks and sugary snacks in the “sugary snacks” pattern. A number of significant associations were found between the respective dietary patterns and the populations’ glycemic and lipidemic profile. However, after adjusting for the overall adjusted threshold, a smaller number of significant associations remained observed. 

The predominant pattern in both populations (the “western breakfast” pattern) appears to relate to food groups whose consumption is primarily found in the basis of a western-type diet [40], such as cheese, processed meat and food items high in carbohydrates (breads and flours for the French). The “western breakfast” pattern reflects a higher percentage (15.61%) of the variance explained in the Greek population, in comparison to the French one (10.58%). This could be explained by the increasing influences of the westernized world trends in the Greek socio-economic scene during the late 2000s. Moreover, breakfast habits were also highlighted in the first 5-year follow-up in the STANISLAS Cohort, which underlined the importance of the household environment in dietary habits by finding a household variance of 42.5 to 52.9% in the energy intake observed in breakfast [29]. The importance of breakfast consumption and its contribution to daily energy intake of French children and families, is also supported by another, recent cross-sectional survey [41]. 

Although the western diet has been associated with elevated inflammation biomarkers [42], the cohort of the Greek teenagers reported no comorbidities and we found no associations between adherence to the “western breakfast” pattern and respective CRP levels. Interestingly enough, the “chicken and sugars” pattern identified in the Greek cohort was significantly associated with lower levels of logCRP (Model 1: β = −0.051, *p*-value = 0.006, Model 2: β = −0.057, *p*-value = 0.004 and Model 3: β = −0.050, *p*-value = 0.008). An inverse association between the consumption of poultry and CRP levels in teenagers has previously been reported, in the general context of adherence to the Dietary Approach to Stop Hypertension (DASH) diet regime [43], although a recent umbrella review showed no association between the DASH diet and CRP levels in adults [44]. On the contrary, an inverse association between consumption of sweets and CRP levels is not supported by other studies. In fact, consumption of sugars and especially sugar-sweetened beverages has previously been associated with higher CRP levels in adults [45,46]. In adolescents, a different review has shown a positive association between sugar consumption and CRP [47], whereas another review found greater consumption of sugars by normal weight adolescents in comparison with overweight ones, but did not find any association between sugar consumption and CRP [48]. Α cross-sectional study investigating the relation between food intake and CRP levels in children also found that consumption of milk, citrus, melons and berries displayed associations with lower levels of CRP, potentially due to the general high content of fruits and vegetables in antioxidants and the association of dairy consumption with greater satiety and potential adherence to a generally healthier diet [49]. 

Furthermore, our study found that the “high protein and animal fat” pattern displayed significant associations with higher logtriglyceride and logBMI levels (*p* < 0.01), for French teenagers. The latter is in accordance with various cross-sectional studies that have researched the dietary habits of adolescents and their potential associations to BMI. A study by Gutiérrez-Pliego et al. unveiled three major dietary patterns in a population of 373 Mexican teenagers including a pattern high in refined “unhealthy” products, such as snacks, sugars and sweets, a pattern with high protein/high fat content and a pattern including high consumption of fruits, vegetables, nuts and whole grains. The study found a strong relationship (*p* < 0.01) between higher consumptions of the first two dietary patterns and higher BMI [50]. In the same context, a different study in Northeastern Brazil investigated data from 1247 adolescents. The study identified two dietary patterns, one referring to high consumption of sugars, sweets and cakes, amongst others, and one correlated with high consumption of fruits and vegetables. Higher adherence to the dietary pattern including “unhealthy” products, was, again, positively correlated with higher values of BMI (*p* = 0.018) [51]. Furthermore, a different study on the dietary habits of female adolescents showed that higher adherence to a “Western” pattern referring to increased consumption of fat and mediocre consumption of protein, among others, was associated with higher levels of BMI, waist circumference, as well as total cholesterol levels [52]. 

Although dietary patterns with a higher consumption of fat have generally been positively associated with cardiometabolic risk factors in teenagers [53,54], certain diets, including consumption of specific food groups, such as the DASH diet [55], have been related with a better metabolic profile [56]. Indeed, higher adherence to the DASH diet has been shown to relate to a reduced prevalence of metabolic syndrome and increased blood pressure during adolescence [43], as well as lower levels of HbA1c and systolic blood pressure, in young adults with type 1 and type 2 diabetes, respectively [57]. Better adherence to the components of the DASH diet was even associated with a lower risk of being a metabolic unhealthy obese, in children and adolescents with increased body weight [58]. Additionally, other high protein diets, such as the ketogenic diet and the Modified Atkins diet, have been associated with better effects on adolescents with epilepsy [59,60], with the ketogenic diet to have been related to reduced weight and fasting insulin and HOMA-IR levels in obese teenagers [61]. However, the aforementioned diets also usually include consumption of vegetables fats and fats derived from nuts, seeds, white meat (such as poultry and fish), as well as food groups like grains, vegetable fats, fruits and vegetables, which are not met when referring to dietary patterns centred on high protein or animal fat consumption. Furthermore, the aforementioned beneficial associations have been primarily observed in adults or obese adolescent populations, who could potentially benefit from the adherence to a structured diet with the above food groups. This could potentially explain why our study demonstrated positive associations between the high consumption of protein and animal fats with BMI and triglyceride levels in adolescents mostly displaying BMI of a normal range. Moreover, the present study evaluates the adherence to each dietary pattern, without comparing them with the respective adherence to the rest of the patterns extracted. 

The identification of dietary patterns of adolescents has generally been a subject of interest in recent literature. Gonzalez-Gil et al. investigated the dietary patterns of 5328 European adolescents in the context of the cross-sectional HELENA study [62]. The latter consisted of adolescent cohorts of 10 different European countries, including Greek teenagers from the cities of Athens and Heraklion, Crete. The study identified four dietary patterns in teenage boys and six dietary patterns in teenage girls. Patterns explaining greater total variance in boys referred to consumption of vegetables, pasta, rice, cheese and sweets among others, at the same time as dominant patterns in girls referred to consumption of Mediterranean-type food items, dairy and consumption of a healthy breakfast [62]. 

Additionally, when investigating the dietary habits of adolescents based on data collected in the 1995 Australian National Nutrition Survey, McNaughton et al. showed that a dietary pattern rich in fruit, salads, cereals and fish was found to be negatively associated with levels of diastolic blood pressure in teenagers older than 16 years of age [63]. Our study found no associations between the patterns containing fruit, vegetable and fish consumption and the levels of diastolic pressure in adolescents younger than 16 years of age.

Furthermore, the I. Family Study investigated the association between the dietary patterns of 2451 pairs of European children and their parents, with regards to the existing food environment conditions. The study showed the role of food availability in the children’s dietary choices, highlighting that the consumption of soft drinks was greatly dependent on their availability in the immediate food environment [64]. Moreno LA et al. also showed that increased consumption of sweet beverages was also associated with increased risk of adolescent obesity [65]. In our study, the “sugary snacks” pattern of the French population, which included consumption of sweetened beverages, was not related to logBMI values, but was associated with lower values of logHDL-C. However the effect disappeared when taking into account the adjusted threshold of statistical significance (0.04 > 0.01). A different study of German adolescents demonstrated that higher consumption of dietary patterns containing high-fat and high-carbohydrate, energy-dense foods was associated with lower socioeconomic levels and a lower intake of various vitamins and minerals [66]. 

A previous publication on the Greek adolescents of the TEENAGE study investigated a spectrum of factors potentially contributing to the development of overweight, leading to the creation of an Overweight Preventive Score, which included breakfast intake, family meals and consumption of sugar-sweetened beverages, among other factors, and further supports the aforementioned findings. The score was found to be significantly associated with a lower likelihood of overweight presence and better levels of glycemic control [67]. 

The limitations of the present study are summarized in the following: (a) data for both populations were collected in a cross-sectional manner, limiting the potential for generalized cause and effect conclusions to be drawn; (b) use of the PCA for the dietary patterns’ extraction, including subjective choices regarding the amount of food groups that are included in the analysis, as well as the number of components to be drawn; (c) comparisons between the two populations’ dietary habits might be affected by the different socio-economic conditions existing in the two countries during the mid-1990s for the STANISLAS and late 2000s for the TEENAGE study. This prolonged gap between the two baseline data collections might manifest itself in the Greek teenagers’ dietary habits, which could have potentially been affected by social changes and changes in food availability and accessibility, mediated by the growing social and technological advancements taking place throughout the 15-year gap. 

## 5. Conclusions

Our study focused on the dietary habits of European adolescents and their potential influence on blood pressure, glycemic and lipidemic profile and inflammation levels. The patterns identified demonstrated associations with indices, such as BMI, and biomarkers, such as triglycerides and CRP. The relations highlighted in the present study display great interest and enhance the need for further research on the pivotal role of diet in the essential-for-development period of adolescence, as a modifying factor for cardiometabolic risk factor-related disorders, such as obesity, hypertension and type 2 diabetes.

## Figures and Tables

**Table 1:** Anthropometric, biochemical and dietary characteristics of the TEENAGE Study population.

	TEENAGE Study						
	All		Boys		Girls		*p*-Value *
	*n*	Median (IQR)	*n*	Median (IQR)	*n*	Median (IQR)	
Age (years)	766	13.30 (1.31)	349	13.36 (1.38)	417	13.26 (1.25)	<0.001
Weight (kg)	766	55.00 (14.00)	349	56.00 (16.00)	417	54.00 (13.00)	0.001
Body Mass Index (BMI) (kg/m^2^)	766	20.88 (4.38)	349	20.85 (4.45)	417	20.93 (4.37)	0.517
Waist-to-hip ratio (WHR)	763	0.76 (0)	349	0.79 (0)	414	0.73 (0)	<0001
Systolic Blood Pressure (SBP) (mmHg)	743	119.00 (16)	335	120.67 (11.93) **	408	118.00 (15)	0.001
Diastolic Blood Pressure (DBP) (mmHg)	743	70.00 (12)	335	71.00 (12)	408	70.00 (12)	0.825
Energy Intake (kcal/day)	766	1741.00 (760)	349	1939.00 (779)	417	1574.00 (609)	<0.001
Glucose (mg/dL),	611	80.00 (12)	283	81.00 (11)	328	79.00 (12)	<0.001
HOMA-IR	539	2.28 (2)	255	2.12 (2)	284	2.37 (2)	<0.001
Insulin (mg/dL)	539	11.00 (7)	255	10.00 (7)	284	12.00 (8)	<0.001
Total Cholesterol (mg/dL)	611	157.00 (33)	283	156.49 (25.18) **	328	157.50 (31)	0.210
Low density lipoprotein Cholesterol (LDL-C) (mg/dL)	611	54.00 (16)	283	90.57 (21.78) **	328	88.40 (26)	0.651
High Density Lipoprotein Cholestrol (HDL- C) (mg/dL)	611	89.20 (27)	283	53.00 (16)	328	56.00 (17)	0.001
Triglycerides (mg/dL)	611	56.00 (24)	283	55.00 (25)	328	57.00 (24)	0.090
C-reactive protein (CRP) (mg/dL)	540	0.30 (1)	254	0.45 (1)	286	0.20 (0)	<0.001

* All hypothesis testing took place via use of the Mann–Whitney test. ** Variable follows the normal distribution and is presented as mean ± sd.

**Table 2:** Anthropometric, biochemical and dietary characteristics of the STANISLAS Family Study population.

	STANISLAS Family Study						
	All		Boys		Girls		*p*-Value *
	*n*	Median (IQR)	*n*	Median (IQR)	*n*	Median (IQR)	
Age (years)	287	13.08 (2.92)	137	13.08 (2.92)	150	13.08 (2.85)	0.416
Weight (kg)	263	46.59 (18.10)	129	47.20 (21.90)	134	46.05 (14.84)	0.136
Body Mass Index (BMI) (kg/m^2^)	263	18.44 (3.61)	129	18.30 (3.20)	134	18.52 (4.18)	0.853
WHR	221	0.77 (0.04) **	110	0.81 (0.03) **	111	0.75 (0.06)	<0.001
Systolic Blood Pressure (SBP) (mmHg)	263	112.00 (14.50)	129	115.60 (11.53) **	134	110.46 (8.76) **	<0.001
Diastolic Blood Pressure (DBP) (mmHg)	263	57.00 (15.50)	129	56.69 (16.00) **	134	57.02 (10.23) **	0.829
Energy Intake (kcal/d)	287	2056.03 (662.24)	137	2070.99 (495.20) **	150	2094.92 (681.16)	0.469
Glucose (mg/dL),	263	88.28 (6.12) **	129	89.18 (6.48) **	134	87.38 (5.76) **	0.018 ***
Total Cholesterol, (mg/dL)	263	179.15 (40.93)	129	173.36 (30.89) **	134	183.01 (36.29)	0.002
Low density lipoprotein cholesterol (LDL-C) (mg/dL)	263	116.99 (33.98)	129	113.13 (28.19) **	134	120.85 (32.05)	0.030
High density lipoprotein cholesterol (HDL-C)(mg/dL)	263	54.05 (20.08)	129	54.44 (15.44) **	134	156.37 (16.99)	0.222
Triglycerides (mg/dL)	263	51.33 (33.63)	129	52.21 (38.05)	134	46.56 (30.09)	0.930
C-reactive protein (CRP) (mg/L)	243	0.30 (0.53)	118	0.32 (0.54)	125	0.26 (0.55)	0.765

* Hypothesis testing took place via use of the Mann–Whitney test wherever at least one variable did not follow the normal distribution. ** Variable follows the normal distribution and is presented as mean ± sd. *** Hypothesis testing took place via the Student’s Independent Samples *t*-test.

**Table 3:** Principal Components Analysis’ factor loadings for the 15 food groups in the TEENAGE study (*n* = 766).

	Component				
Food Groups	1	2	3	4	5
Cheese	0.897	-	-	-	-
Dairy	0.863	-	-	-	-
Processed Meat	0.635	-	-	-	-
Legumes	-	0.739	-	-	-
Olives, Olive Oil, Nuts	-	0.668	-	-	-
Red Meat	-	-	0.712	−0.429	-
Potatoes	-	-	0.661	-	-
Fish	-	−0.358	−0.480	-	-
Chicken	-	-	-	0.649	-
Sweets	-	-	-	0.518	-
Fruit and Juices	-	-	-	−0.368	-
Non-refined cereals	-	-	-	-	0.674
Vegetables	-	-	-	-	0.342
Eggs	-	-	-	-	0.303
Refined Cereals	0.512	-	-	-	−0.595
Total Variance Explained (%)	15.61	10.32	8.33	7.60	7.47

Only loadings with an absolute values > 0.3 are presented in the table.

**Table 4:** Principal Components Analysis’ factor loadings for the 15 food groups in the STANISLAS Family study. (*n* = 287).

	Component				
Food Groups	1	2	3	4	5
Cheese	0.664	-	-	-	-
Breads and Flours	0.605	-	-	-	-
Processed Meat	0.523	-	-	-	-
Vegetables	0.483	-	-	-	-
Eggs	-	0.630	-	-	-
Salty Snacks	-	−0.580	-	-	-
Vegetable Fat	-	0.576	-	-	-
Red Meat	-	-	0.703	-	-
Animal Fat	-	-	0.610	-	-
Milk and Yogurt	-	-	0.473	−0.338	-
Fish	-	-	-	0.666	-
Seafood	-	-	-	0.628	-
Poultry	-	-	-	−0.380	-
Soft Drinks	-	-	-	-	0.777
Sugars, Sweets and Cereal Bars	-	-	-	-	0.746
Total Variance Explained (%)	10.58	10.44	9.26	8.19	8.19

Only loadings with an absolute values > 0.3 are presented in the table.

**Table 5:** Linear Regression Analyses on the association between the dietary patterns, anthropometric indices and biomarkers of glycemic and lipidemic control in the TEENAGE study.

	Model 1			Model 2			Model 3			Model 4		
	β	SE	*p*	β	SE	*p*	β	SE	*p*	β	SE	*p*
**LogBMI**												
Western Breakfast	−0.004	0.003	0.150	−0.003	0.003	0.308	-	-	-	-	-	-
Legumes and Good Fat	−0.006	0.003	0.017	−0.004	0.003	0.194	-	-	-	-	-	-
Homemade Meal	−0.005	0.003	0.042	−0.003	0.003	0.242	-	-	-	-	-	-
Chicken and Sugars	−0.005	0.003	0.069	−0.004	0.003	0.128	-	-	-	-	-	-
Eggs and Fibers	0.004	0.003	0.111	0.004	0.003	0.115	-	-	-	-	-	-
**LogWHR**												
Western Breakfast	0.013	0.012	0.270	0.016	0.13	0.247	0.017	0.013	0.198	0.017	0.014	0.250
Legumes and Good Fat	−0.006	0.011	0.622	−0.008	0.013	0.527	−0.007	0.013	0.608	−0.007	0.013	0.597
Homemade Meal	−0.009	0.011	0.445	−0.008	0.013	0.517	−0.007	0.013	0.599	−0.008	0.013	0.562
Chicken and Sugars	−0.003	0.011	0.760	−0.005	0.013	0.696	−0.003	0.013	0.828	−0.003	0.013	0.800
Eggs and Fibers	−0.011	0.011	0.320	−0.0013	0.013	0.339	−0.015	0.013	0.268	−0.015	0.013	0.267
**LogSBP**												
Western Breakfast	−0.003	0.002	0.085	−0.002	0.002	0.174	−0.002	0.002	0.295	−0.001	0.002	0.646
Legumes and Good Fat	0.000	0.002	0.838	0.001	0.002	0.729	0.001	0.002	0.499	0.001	0.002	0.472
Homemade Meal	0.000	0.002	0.937	0.000	0.002	0.819	0.001	0.002	0.579	0.001	0.002	0.481
Chicken and Sugars	0.002	0.002	0.169	0.002	0.002	0.246	0.003	0.002	0.090	0.003	0.002	0.071
Eggs and Fibers	2.294 × 10^−5^	0.002	0.988	−0.001	0.002	0.680	−0.001	0.002	0.409	−0.001	0.002	0.411
**LogDBP**												
Western Breakfast	−0.003	0.002	0.224	−0.003	0.002	0.256	−0.002	0.002	0.361	0.000	0.003	0.894
Legumes and Good Fat	−0.002	0.002	0.482	−0.001	0.002	0.786	0.000	0.002	0.948	−3.047 × 10^−5^	0.002	0.990
Homemade Meal	0.001	0.002	0.551	0.003	0.002	0.155	0.004	0.002	0.097	0.004	0.002	0.063
Chicken and Sugars	0.001	0.002	0.609	0.001	0.002	0.528	0.002	0.002	0.333	0.003	0.002	0.271
Eggs and Fibers	0.001	0.002	0.802	0.000	0.002	0.878	0.000	0.002	0.914	0.000	0.002	0.919
**LogGlucose**												
Western Breakfast	−0.003	0.007	0.655	−0.003	0.007	0.632	−0.003	0.007	0.631	−0.004	0.008	0.615
Legumes and Good Fat	0.010	0.006	0.120	0.011	0.007	0.111	0.011	0.007	0.110	0.011	0.007	0.111
Homemade Meal	−0.002	0.006	0.740	−0.004	0.007	0.531	−0.004	0.007	0.531	−0.004	0.007	0.532
Chicken and Sugars	0.015	0.006	0.017	0.013	0.007	0.051	0.013	0.007	0.051	0.013	0.007	0.051
Eggs and Fibers	0.003	0.006	0.588	0.003	0.007	0.659	0.003	0.007	0.659	0.003	0.007	0.660
**LogInsulin**												
Western Breakfast	−0.015	0.010	0.119	−0.015	0.010	0.139	−0.009	0.010	0.356	−0.007	0.010	0.521
Legumes and Good Fat	−0.020	0.009	0.030	−0.019	0.010	0.066	−0.017	0.009	0.066	−0.017	0.009	0.064
Homemade Meal	0.011	0.010	0.247	0.011	0.010	0.250	0.013	0.009	0.167	0.014	0.009	0.142
Chicken and Sugars	0.012	0.009	0.191	0.013	0.010	0.173	0.018	0.009	0.049	0.018	0.009	0.041
Eggs and Fibers	−0.015	0.009	0.113	−0.011	0.010	0.281	−0.014	0.010	0.133	−0.014	0.010	0.132
**LogHOMA-IR**												
Western Breakfast	−0.016	0.011	0.158	−0.016	0.012	0.180	−0.035	0.011	0.422	−0.004	0.012	0.728
Legumes and Good Fat	−0.020	0.010	0.054	−0.020	0.011	0.074	−0.019	0.011	0.075	−0.019	0.011	0.072
Homemade Meal	0.014	0.011	0.205	0.013	0.011	0.231	0.015	0.010	0.157	0.016	0.010	0.124
Chicken and Sugars	0.010	0.010	0.349	0.010	0.011	0.345	0.015	0.010	0.139	0.016	0.010	0.114
Eggs and Fibers	−0.018	0.010	0.089	−0.017	0.012	0.157	−0.020	0.011	0.067	−0.020	0.011	0.066
**LogTotalCholesterol**												
Western Breakfast	−0.005	0.003	0.066	−0.006	0.003	0.060	−0.006	0.003	0.054	−0.003	0.003	0.422
Legumes and Good Fat	0.001	0.003	0.721	0.001	0.003	0.863	0.000	0.003	0.883	0.000	0.003	0.908
Homemade Meal	0.002	0.003	0.402	0.002	0.003	0.538	0.002	0.003	0.549	0.003	0.003	0.353
Chicken and Sugars	0.000	0.003	0.917	2.502 × 10^−^^5^	0.003	0.993	−5.600 × 10^−5^	0.003	0.985	0.000	0.003	0.868
Eggs and Fibers	0.003	0.003	0.269	0.002	0.003	0.521	0.002	0.003	0.511	0.002	0.003	0.511
**LogHDL-C**												
Western Breakfast	−0.002	0.004	0.553	−0.002	0.004	0.692	−0.004	0.004	0.313	−0.002	0.005	0.643
Legumes and Good Fat	0.006	0.004	0.160	0.005	0.004	0.210	0.004	0.004	0.343	0.004	0.004	0.351
Homemade Meal	0.001	0.004	0.832	0.001	0.004	0.900	0.000	0.004	0.919	0.000	0.004	0.958
Chicken and Sugars	0.009	0.004	0.022	0.007	0.004	0.080	0.006	0.004	0.153	0.006	0.004	0.128
Eggs and Fibers	−0.001	0.004	0.885	−0.002	0.004	0.600	−0.001	0.004	0.761	−0.001	0.004	0.759
**LogLDL-C**												
Western Breakfast	−0.008	0.005	0.099	−0.009	0.005	0.053	−0.009	0.005	0.073	−0.004	0.005	0.460
Legumes and Good Fat	−0.001	0.004	0.761	−0.003	0.005	0.547	−0.002	0.005	0.610	−0.003	0.005	0.586
Homemade Meal	0.003	0.004	0.566	0.001	0.005	0.800	0.001	0.005	0.753	0.003	0.005	0.537
Chicken and Sugars	−0.005	0.004	0.246	−0.005	0.005	0.278	−0.004	0.005	0.324	−0.004	0.004	0.411
Eggs and Fibers	0.005	0.004	0.233	0.004	0.005	0.389	0.004	0.005	0.423	0.004	0.005	0.423
**LogTriglycerides**												
Western Breakfast	−0.003	0.006	0.632	0.002	0.007	0.747	0.001	0.006	0.831	0.004	0.007	0.573
Legumes and Good Fat	0.006	0.006	0.307	0.008	0.006	0.208	0.010	0.006	0.101	0.010	0.006	0.103
Homemade Meal	−0.005	0.006	0.441	−0.004	0.006	0.550	−0.002	0.006	0.686	−0.002	0.006	0.745
Chicken and Sugars	−0.006	0.006	0.329	−0.004	0.006	0.491	−0.002	0.006	0.728	−0.002	0.006	0.764
Eggs and Fibers	−0.002	0.006	0.776	−0.005	0.007	0.418	−0.007	0.006	0.288	−0.007	0.006	0.287
**LogCRP**												
Western Breakfast	0.002	0.020	0.939	0.006	0.021	0.775	0.018	0.020	0.383	0.021	0.022	0.349
Legumes and Good Fat	0.006	0.019	0.759	0.019	0.021	0.369	0.022	0.020	0.275	0.022	0.020	0.276
Homemade Meal	0.015	0.020	0.444	0.005	0.021	0.795	0.007	0.019	0.714	0.007	0.020	0.714
Chicken and Sugars	−0.051	0.019	0.006	−0.057	0.020	0.004	−0.050	0.019	0.008	−0.051	0.019	0.008
Eggs and Fibers	0.016	0.019	0.418	0.029	0.021	0.175	0.023	0.020	0.266	0.023	0.020	0.266

Model 1: Adjusted for age and sex; Model 2: Adjusted for age, sex, physical activity; Model 3: Adjusted for age, sex, physical activity, BMI; Model 4: Adjusted for age, sex, physical activity, BMI, energy intake.

**Table 6:** Linear mixed model analyses on the association between the dietary patterns, anthropometric indices and biomarkers of glycemic and lipidemic control in the STANISLAS Family study.

	Model 1			Model 2			Model 3			Model 4		
	Estimate	SE	*p*	Estimate	SE	*p*	Estimate	SE	*p*	Estimate	SE	*p*
**LogBMI**												
Western Breakfast	0.000	0.003	0.878	0.000	0.005	0.459	-	-	-	-	-	-
Prudent Snacking	0.000	0.003	0.950	0.001	0.003	0.738	-	-	-	-	-	-
High Protein and Animal Fat	0.011	0.003	0.002	0.009	0.003	0.018	-	-	-	-	-	-
Fish and Seafood	−0.002	0.003	0.430	−0.001	0.003	0.700	-	-	-	-	-	-
Sugary Snacks	−0.001	0.003	0.701	−0.002	0.003	0.437	-	-	-	-	-	-
**LogWHR**												
Western Breakfast	−0.000	0.001	0.800	−0.000	0.001	0.539	−0.000	0.001	0.540	−0.000	0.001	0.840
Prudent Snacking	3.965729 × 10^−5^	0.001	0.976	0.000	0.001	0.809	0.000	0.001	0.797	0.000	0.001	0.722
High protein and animal Fat	0.000	0.001	0.723	0.000	0.001	0.616	0.000	0.001	0.757	0.001	0.001	0.486
Fish and Seafood	0.001	0.001	0.134	0.002	0.001	0.146	0.002	0.001	0.126	0.002	0.001	0.130
Sugary Snacks	−0.001	0.001	0.392	−0.001	0.001	0.363	−0.001	0.001	0.409	−0.000	0.001	0.691
**LogSBP**												
Western Breakfast	−2.288744 × 10^−5^	0.002	0.991	0.000	0.002	0.892	0.000	0.002	0.837	−0.000	0.002	0.792
Prudent Snacking	0.003	0.002	0.114	0.003	0.002	0.181	0.002	0.002	0.189	0.002	0.002	0.215
High protein and Animal Fat	0.000	0.002	0.733	0.000	0.002	0.822	−0.000	0.002	0.802	−0.001	0.002	0.504
Fish and Seafood	−0.000	0.002	0.751	−0.000	0.002	0.766	−0.000	0.002	0.801	−0.000	0.002	0.794
Sugary Snacks	0.000	0.002	0.640	0.000	0.002	0.787	0.000	0.002	0.673	−0.000	0.002	0.894
**LogDBP**												
Western Breakfast	−0.000	0.004	0.948	0.003	0.004	0.510	0.003	0.004	0.483	0.003	0.005	0.464
Prudent Snacking	0.002	0.004	0.593	0.001	0.004	0.833	0.000	0.004	0.841	0.000	0.004	0.845
High Protein and Animal Fat	−0.008	0.004	0.089	−0.008	0.005	0.099	−0.010	0.005	0.045	−0.012	0.005	0.028
Fish and Seafood	0.009	0.004	0.039	0.008	0.004	0.077	0.008	0.004	0.069	0.008	0.004	0.070
Sugary Snacks	−0.000	0.004	0.936	−0.002	0.005	0.651	−0.001	0.005	0.718	−0.002	0.006	0.632
**LogGlucose**												
Western Breakfast	0.000	0.001	0.604	0.001	0.002	0.448	0.001	0.002	0.462	0.000	0.002	0.868
Prudent Snacking	−0.000	0.001	0.917	−0.000	0.002	0.793	−0.000	0.002	0.805	−0.000	0.002	0.727
High Protein and Animal Fat	−0.001	0.002	0.428	−0.001	0.002	0.632	−0.000	0.002	0.708	−0.002	0.002	0.365
Fish and Seafood	−0.002	0.001	0.202	−0.001	0.001	0.331	−0.001	0.001	0.323	−0.001	0.001	0.323
Sugary Snacks	0.001	0.001	0.568	0.000	0.002	0.906	0.000	0.002	0.928	−0.001	0.002	0.502
**LogTotalCholesterol**												
Western Breakfast	−0.001	0.004	0.728	−0.002	0.004	0.644	−0.002	0.004	0.66	−0.002	0.005	0.703
Prudent Snacking	0.002	0.004	0.599	0.004	0.004	0.347	0.004	0.004	0.369	0.004	0.004	0.358
High Protein and Animal Fat	−0.003	0.005	0.490	−0.006	0.005	0.236	−0.007	0.005	0.157	−0.008	0.005	0.151
Fish and Seafood	0.005	0.004	0.224	0.006	0.004	0.173	0.006	0.004	0.171	0.006	0.004	0.172
Sugary Snacks	−0.001	0.004	0.712	6.925668 × 10^−7^	0.005	1.000	0.000	0.005	0.940	0.001	0.006	0.833
**LogHDL-C**												
Western Breakfast	0.006	0.006	0.303	0.005	0.007	0.426	0.005	0.007	0.443	0.011	0.007	0.139
Prudent Snacking	−0.005	0.006	0.419	−0.004	0.007	0.547	−0.003	0.007	0.584	−0.003	0.007	0.657
High Protein and Animal Fat	−0.003	0.007	0.621	−0.002	0.008	0.762	0.000	0.008	0.983	0.004	0.008	0.622
Fish and Seafood	0.004	0.006	0.462	0.002	0.006	0.710	0.002	0.006	0.728	0.002	0.006	0.746
Sugary Snacks	−0.007	0.006	0.237	−0.014	0.007	0.065	−0.014	0.007	0.049	−0.013	0.008	0.114
**LogLDL-C**												
Western Breakfast	−0.006	0.006	0.333	−0.007	0.006	0.275	−0.007	0.006	0.292	−0.060	0.053	0.254
Prudent Snacking	0.004	0.006	0.493	0.007	0.006	0.293	0.006	0.006	0.332	0.041	0.047	0.391
High Protein and Animal Fat	−0.005	0.007	0.472	−0.010	0.007	0.168	−0.013	0.007	0.073	−0.112	0.057	0.050
Fish and Seafood	0.004	0.006	0.475	0.007	0.006	0.292	0.006	0.006	0.288	0.035	0.045	0.435
Sugary Snacks	−0.001	0.006	0.810	0.005	0.007	0.492	0.005	0.007	0.410	0.042	0.059	0.473
**LogTriglycerides**												
Western Breakfast	0.011	0.012	0.338	0.009	0.013	0.467	0.010	0.013	0.444	−0.001	0.014	0.911
Prudent Snacking	0.003	0.012	0.237	0.000	0.013	0.990	−6.768397 × 10^−5^	0.013	0.996	−0.001	0.013	0.893
High Protein and Animal Fat	0.054	0.013	<0.001	0.049	0.014	0.001	0.045	0.014	0.002	0.041	0.015	0.009
Fish and Seafood	0.014	0.012	0.252	0.019	0.012	0.133	0.020	0.012	0.114	0.021	0.012	0.093
Sugary Snacks	0.009	0.012	0.428	0.010	0.013	0.462	0.011	0.013	0.399	−0.002	0.016	0.855
**LogCRP**												
Western Breakfast	−0.045	0.029	0.125	−0.053	0.031	0.085	−0.050	0.030	0.096	−0.076	0.033	0.024
Prudent Snacking	0.031	0.028	0.274	0.037	0.030	0.217	0.037	0.029	0.201	0.036	0.029	0.222
High Protein and Animal Fat	0.009	0.031	0.757	−0.005	0.033	0.873	−0.019	0.032	0.558	−0.033	0.034	0.334
Fish and Seafood	0.018	0.029	0.516	0.009	0.030	0.745	0.010	0.029	0.733	0.008	0.030	0.774
Sugary Snacks	0.010	0.031	0.743	0.011	0.032	0.729	0.016	0.032	0.603	0.004	0.036	0.905

Model 1: Adjusted for age and sex; Model 2: Adjusted for age, sex, physical exercise; Model 3, Adjusted for age, sex, physical activity, BMI. Original data values in mmol/l were used for creation of the logGlucose, logTotalCholesterol, logHDL-C, logLDL-C, LogTriglycerides variables.

## Data Availability

Data available on request due to restrictions eg privacy or ethical. The data presented in this study are available on request from the corresponding author. The data are not publicly available due to privacy/ethical restrictions on the data provided by the volunteers.

## References

[1] Gidding S.S., Dennison B.A., Birch L.L., Daniels S.R., Gilman M.W., Lichtenstein A.H., Rattay S.R., Steinberg J., Stettler N., American Heart Association (2006). Dietary Recommendations for Children and Adolescents: A Guide for Practitioners. Pediatrics.

[2] World Health Organization (2018). Guideline: Implementing Effective Actions for Improving Adolescent Nutrition. https://apps.who.int/iris/bitstream/handle/10665/260297/9789241513708-eng.pdf?sequence=1.

[3] National Heath System (2018). Healthy Eating for Teens-Eat Well. https://www.nhs.uk/live-well/eat-well/healthy-eating-for-teens/.

[4] (2018). British Nutrition Foundation Teenagers. https://www.nutrition.org.uk/healthyliving/lifestages/teenagers.html?start=2.

[5] DiGirolamo A.M., Ochaeta L., Mejia Flores R.M. (2020). Early Childhood Nutrition and Cognitive Functioning in Childhood and Adolescence. Food Nutr. Bull..

[6] Cohen J.F.W., Gorski M.T., Gruber S.A., Kurdziel L.B.F., Rimm E.B. (2016). The effect of healthy dietary consumption on executive cognitive functioning in children and adolescents: A systematic review. Br. J. Nutr..

[7] Nyaradi A., Foster J.K., Hickling S., Li J., Ambrosini G.L., Jacques A., Oddy W.H. (2014). Prospective associations between dietary patterns and cognitive performance during adolescence. J. Child. Psychol. Psychiatry.

[8] O’Neil A., Quirk S.E., Housden S., Brennan S.L., Williams L.J., Pasco J.A., Berk M., Jacka F.N. (2014). Relationship between diet and mental health in children and adolescents: A systematic review. Am. J. Public Health.

[9] World Health Organization (2017). Adolescent Obesity and Related Behaviours: Trends and Inequalities in the WHO European Region, 2002–2014. http://www.euro.who.int/__data/assets/pdf_file/0019/339211/WHO_ObesityReport_2017_v3.pdf.

[10] World Health Organization (2016). Adolescents’ Dietary Habits. https://www.euro.who.int/__data/assets/pdf_file/0006/303477/HBSCNo.7_factsheet_Diet.pdf%3Fua%3D1.

[11] World Health Organization (2019). United Nations Agency Briefs: Responding to the Challenge of Non-communicable Diseases. https://apps.who.int/iris/bitstream/handle/10665/327396/WHO-UNIATF-19.98-eng.pdf?ua=1.

[12] Suchindran C., North K.E., Popkin B.M., Gordon-Larsen P. (2010). The association of adolescent obesity with risk of severe obesity in adulthood. JAMA.

[13] Biro F.M., Wien M. (2010). Childhood obesity and adult morbidities. Am. J. Clin. Nutr..

[14] Engeland A., Bjørge T., Tverdal A., Søgaard A.J. (2004). Obesity in adolescence and adulthood and the risk of adult mortality. Epidemiology.

[15] Mirmiran P., Ziadlou M., Karimi S., Hosseini-Esfahani F., Azizi F. (2019). The association of dietary patterns and adherence to WHO healthy diet with metabolic syndrome in children and adolescents: Tehran lipid and glucose study. BMC Public Health.

[16] Pulgaron E.R., Delamater A.M. (2014). Oesity and type 2 diabetes in children: Epidemiology and treatment. Curr. Diab. Rep..

[17] Barr M.M., Aslibekyan S., Ashraf A.P. (2019). Glycemic control and lipid outcomes in children and adolescents with type 2 diabetes. PLoS ONE.

[18] Lampropoulou M., Chaini M., Rigopoulos N., Evangeliou A., Papadopoulou-Legbelou K., Koutelidakis A.E. (2020). Association between serum lipid levels in Greek children with dyslipidemia and mediterranean diet adherence, dietary habits, lifestyle and family socioeconomic factors. Nutrients.

[19] Ferreira de Moraes A.C., Lacerda M.B., Moreno L.A., Horta B.L., Carvalho H.B. (2014). Prevalence of high blood pressure in 122,053 adolescents: A systematic review and meta-regression. Medicine.

[20] Ntalla I., Giannakopoulou M., Vlachou P., Giannitsopoulou K., Gkesou V., Makridi C., Marougka M., Mikou G., Ntaoutidou K., Proutzou E. (2014). Body composition and eating behaviours in relation to dieting involvement in a sample of urban Greek adolescents from the TEENAGE (TEENs of Attica: Genes & Environment) study. Public Health Nutr..

[21] Ntalla I., Panoutsopoulou K., Vlachou P., Southam L., Rayner N.W., Zeggini E., Dedoussis G.V. (2013). Replication of established common genetic variants for adult BMI and childhood obesity in Greek Adolescents: The TEENAGE Study. Ann. Hum. Genet..

[22] World Medical Association (2001). World Medical Association Declaration of Helsinki. Ethical principles for medical research involving human subjects. Bull. World Health Organ..

[23] Nutritionist Pro Software. https://www.nutritionistpro.com/.

[24] Schofield W.N. (1985). Predicting basal metabolic rate, new standards and review of previous work. Hum. Nutr. Clin. Nutr..

[25] Food and Agriculture Organization of the United Nations, World Health Organization, United Nations (2004). University Human Energy Requirements: Report of a Joint FAO/WHO/UNU Expert Consultation.

[26] Goldberg G.R., Black A.E., Jebb S.A., Murgatroyd P.R., Cpward W.A., Prentice A.M. (1991). Critical evaluation of energy intake data using fundamental principles of energy physiology: 1. Derivation of cut-off limits to identify under-recording. Eur. J. Clin. Nutr..

[27] Sichert-Hellert W., Kersting M., Schoch G. (1998). Underreporting of energy intake in 1 to 18 year old German children and adolescents. Z. Ernahr..

[28] Siest G., Visvikis S., Herbeth B., Gueguen R., Vincent-Viry M., Sass C., Beaud B., Lecomte E., Steinmetz J., Locuty J. (1998). Objectives, Design and Recruitment of a Familial and Longitudinal Cohort for Studying Gene-Environment Interactions in the Field of Cardiovascular Risk: The Stanislas Cohort. Clin. Chem. Lab. Med..

[29] Billon S., Lluch A., Gueguen R., Berthier A.M., Siest G., Herbeth B. (2002). Family resemblance in breakfast energy intake: The Stanislas Family Study. Eur. J. Clin. Nutr..

[30] Visvikis-Siest S., Siest G. (2008). The STANISLAS Cohort: A 10-year follow-up of supposed healthy families. Gene-environment interactions, reference values and evaluation of biomarkers in prevention of cardiovascular diseases. Clin. Chem. Lab. Med..

[31] Musse N., Michaud C., Musse J.P., Nicolas J.P. Gestion informatisee de l’enquete alimentaire. Proceedings of the XIIeme Congres International de Medecine Sociale.

[32] Krishnaveni R., Gowda V.M.N. (2015). Assessing the validity of Friedewald’s formula and Anandraja’s formula for serum LDL–cholesterol calculation. J. Clin. Diagn. Res..

[33] IBM Support SPSS Statistics 23.0 Now Available for Download. www.Ibm.com. https://www.ibm.com/support/pages/spss-statistics-230-now-available-download.

[34] Jolliffe I.T., Cadima J. (2016). Principal component analysis: A review and recent developments. Philos. Trans. A Math. Phys. Eng. Sci..

[35] Schwedhelm C., Iqbal K., Knuppel S., Schwingshackl L., Boeing H. (2018). Contribution to the understanding of how principal component analysis-derived dietary patterns emerge from habitual data on food consumption. Am. J. Clin. Nutr..

[36] Smith A.D.A.C., Emmett P.M., Newby P., Northstone K. (2013). Dietary patterns obtained through principal components analysis: The effect of input variable quantification. Br. J. Nutr..

[37] Knafl G.J., Dixon J.K., O’Malley J.P., Grey M., Deatrick J.A., Gallo A.M., Knafl K.A. (2009). Analysis of cross-sectional univariate measurements for family dyads using linear mixed modeling. J. Fam. Nurs..

[38] Van Dongen H.P.A., Olofsen E., Dinges D.F., Maislin G. (2004). Mixed-model regression analysis and dealing with interindividual differences. Methods Enzymol..

[39] European Food Safety Authority (EFSA), EFSA Panel on Dietetic Products, Nutrition and Allergies (NDA) (2013). Scientific Opinion on Dietary Reference Values for energy. EFSA J..

[40] Cordain L., Eaton S.B., Sebastian A., Mann N., Lindeberg S., Watkins B.A., O’Keefe J.H., Brand-Miller J. (2005). Origins and evolution of the Western diet: Health implications for the 21st century. Am. J. Clin. Nutr..

[41] Bellisle F., Hebel P., Salmon-Legagneur A., Vieux F. (2018). Breakfast consumption in french children, adolescents, and adults: A nationally representative cross-sectional survey examined in the context of the international Breakfast Research Initiative. Nutrients.

[42] Manzel A., Muller D.N., Hafler D.A., Erdman S.E., Linker R.A., Kleinewietfeld M. (2014). Role of “Western Diet” in Inflammatory Autoimmune Diseases. Curr. Allergy Asthma Rep..

[43] Saneei P., Hashemipour M., Kelishadi R., Esmaillzadeh A. (2014). The dietary approaches to stop hypertension (DASH) diet affects inflammation in childhood metabolic syndrome: A randomized cross-over clinical trial. Ann. Nutr. Metab..

[44] Chiavaroli L., Viguiliouk E., Nish S.K., Blanco Mejia S., Rahelic D., Kahleova H., Salas-Salvado J., Kendall C.W.C., Sievenpiper J.L. (2019). DASH dietary pattern and cardiometabolic outcomes: An umbrella review of systematic reviews and meta-analyses. Nutrients.

[45] Mazidi M., Kengne A.P., Mikhailidis D.P., Cicero A., Banach M. (2018). Effects of selected dietary constituents on high-sensitivity C-reactive protein levels in U.S. adults. Ann. Med..

[46] O’Connor L., Imamura F., Brage S., Griffin S.J., Wareham N.J., Forouhi N.G. (2018). Intakes and sources of dietary sugars and their association with metabolic and inflammatory markers. Clin. Nutr..

[47] Lazarou C., Philippou E., Preedy V.R., Hunter L.A., Patel V.B. (2013). C-reactiive protein and diet quality in Children. Diet Quality: An Evidence-Based Approach.

[48] Karampola M., Argiriou A., Hitoglou-Makedou A. (2019). Study on dietary constituents, hs-CRP serum levels and investigation of correlation between them in excess weight adolescents. Hippokratia.

[49] Qureshi M.M., Singer M.R., Moore L.L. (2009). A cross-sectional study of food group intake and C-reactive protein among children. Nutr. Meta (Lond.).

[50] Gutiérrez-Pliego L.E., Camarillo-Romero E.S., Montenegro-Morales L.P., Garduño-García J.D.J. (2016). Dietary patterns associated with body mass index (BMI) and lifestyle in Mexican adolescents. BMC Public Health.

[51] Santos N.H., Fiaccone R.L., Barreto M.L., Silva L.A.D., Silva R.D.C.R. (2014). Association between eating patterns and body mass index in a sample of children and adolescents in Northeastern Brazil. Cad. Saúde Pública.

[52] Ambrosini G.L., Huang R.C., Mori T.A., Hands B.P., O’Sullivan T.A., de Klerk N., Beilin L.J., Oddy W.H. (2010). Dietary patterns and markers for the metabolic syndrome in Australian adolescents. Nutr. Metab. Cardiovasc. Dis..

[53] Rocha N.P., Cupertino L.M., Longo G.Z., Ribeiro A.Q., Novaes J.F. (2017). Association between dietary pattern and cardiometabolic risk in children and adolescents: A systematic review. J. Pediatr..

[54] Joung H., Hong S., Song Y., Ahn B.C., Park M.J. (2012). Dietary patterns and metabolic syndrome risk factors among adolescents. Korean J. Pediatr..

[55] National Heart, Lung, and Blood Institute (2009). DASH Eating Plan. http://www.nhlbi.nih.gov/health/resources/heart/hbp-dash-introduction-html.

[56] Castro-Barquero S., Ruiz-León A.M., Pérez-Sierra M., Estruch R., Casas R. (2020). Dietary strategies for metabolic syndrome: A comprehensive review. Nutrients.

[57] Barnes T.L., Crandell J.L., Bell R.A., Mayer-Davis E.J., Dabelea D., Liese A.D. (2013). Change in DASH diet score and cardiovascular risk factors in youth with type 1 and type 2 diabetes mellitus: The SEARCH for Diabetes in Youth Study. Nutr. Diabetes.

[58] Rahimi H., Yuzbashian E., Zareie R., Asghari G., Djazayery A., Movahedi A., Mirmiran P. (2020). Dietary approaches to stop hypertension (DASH) score and obesity phenotypes in children and adolescents. Nutr. J..

[59] Wells J., Swaminathan A., Paseka J., Hanson C. (2020). Efficacy and safety of a ketogenic diet in children and adolescents with refractory epilepsy—A review. Nutrients.

[60] Payne N.E., Cross H., Sander J.W., Sisodiya S.M. (2011). The ketogenic and related diets in adolescents and adults—A review. Epilepsia.

[61] Partsalaki I., Karvela A., Spiliotis B.E. (2012). Metabolic impact of a ketogenic diet compared to a hypocaloric diet in obese children and adolescents. J. Pediatr. Endocr. Met..

[62] González-Gil E.M., Martínez-Olivan B., Widhalm K., Lambrinou C.P., De Henauw S., Gottrand F., Kafatos A., Beghin L., Molnar D., Kersting M. (2019). Healthy eating determinants and dietary patterns in European adolescents: The HELENA study. Child Adolesc. Obes..

[63] McNaughton S.A., Ball K., Mishra G.D., Crawford D.A. (2008). Dietary patterns of adolescents and risk of obesity and hypertension. J. Nutr..

[64] Hebestreit A., Intemann T., Siani A., De Henauw S., Eibenm G., Kourides Y., Kovacs E., Moreno L.A., Veidebaum T. (2017). Dietary patterns of european children and their parents in association with family food environment: Results from the I. family study. Nutrients.

[65] Moreno L.A., Rodriguez G., Fieta J., Bueno-Lozano M., Lázaro A., Bueno G. (2010). critical reviews in food science and nutrition. Crit. Rev. Food. Sci. Nutr..

[66] Richter A., Heidemann C., Schulze M.B., Roosen J., Thiele S., Mensink G.B.M. (2012). Dietary patterns of adolescents in Germany—Associations with nutrient intake and other health related lifestyle characteristics. BMC Pediatr..

[67] Ntalla I., Yannaoulia M., Dedoussis G.V. (2016). An overweight preventive score associates with obesity and glycemic traits. Metabolism.

